# Higher thyroid hormone has a negative association with lower limb lean body mass in euthyroid older adults: Analysis from the Baltimore Longitudinal study of aging

**DOI:** 10.3389/fragi.2023.1150645

**Published:** 2023-04-11

**Authors:** Hamza Ahmed Ibad, Jennifer S. Mammen, Eleanor M. Simonsick, C. Kent Kwoh, Ali Guermazi, Shadpour Demehri

**Affiliations:** ^1^ The Russell H. Morgan Department of Radiology and Radiological Science, Johns Hopkins University School of Medicine, Baltimore, MD, United States; ^2^ The Department of Medicine, Johns Hopkins University School of Medicine, Baltimore, MD, United States; ^3^ National Institute of Aging, NIH, Baltimore, MD, United States; ^4^ Division of Rheumatology, The University of Arizona, Tucson, AZ, United States; ^5^ Department of Radiology, Boston University School of Medicine, Boston, MA, United States

**Keywords:** thyroid hormone, DEXA, duel-energy X-ray absorptiometry, sarcopenia, older adults, muscle mass and fat mass

## Abstract

**Background:** Hyperthyroidism is associated with lower lean body mass, as a result of catabolic actions of thyroid hormone. Therefore, higher thyroid hormone levels could be a factor in the development of sarcopenia and age associated functional decline. The relationship between thyroid hormone and muscle mass in ambulatory, euthyroid older adults is not known.

**Method:** We used mixed-effects models to estimate the cross-sectional relationships (accounting for inter-person variability) between thyroid axis hormone measures and lower limb composition or sarcopenia at visits in the Baltimore Longitudinal Study of Aging (BLSA) at which DEXA scans were available and both thyrotropin (TSH) and free thyroxine (FT4) were in the reference range. Analyses were adjusted for levothyroxine use, age, race, sex, BMI, smoking, alcohol intake, cholesterol, and systolic blood pressure.

**Results:** 1442 euthyroid participants (median age 68, 50% female, and 69% white) contributed to 5306 visits from 2003 to 2019. FT4 was negatively associated with lower limb lean mass (beta: 88.49; 95% Confidence Interval (CI): 122.78, −54.20; *p* < 0.001) and positively associated with sarcopenia (OR: 1.11%, 95% CI: 1.01, 1.22) in the whole cohort. Additionally, higher FT4 was associated with lower leg lean mass (beta: 66.79; 95% CI: 102.24, −31.33; *p* < 0.001) and sarcopenia (OR:1.09%, 95% CI:1.01, 1.18) in older adults, but not in younger adults alone.

**Conclusion:** In euthyroid older adults, higher FT4 is associated with lower leg lean mass and higher odds of sarcopenia. Understanding the relationship between thyroid hormone and sarcopenia is needed to improve clinical decision-making and avoid functional decline from excess thyroid hormone use in older adults.

## Introduction

Sarcopenia, the loss of skeletal muscle mass and function with age, (Cruz-Jentoft et al., 2019), been associated with adverse outcomes such as falls, fracture (Yeung et al., 2019) and mortality. (Brown et al., 2016). Thyroid hormone has catabolic effects on muscle and therefore represents a potentially modifiable risk factor for age related muscle loss. That hyperthyroidism leads to a loss of muscle mass and strength is well documented (Brennan et al., 2006) but studies of the relationship between thyroid hormone levels and muscle mass in euthyroid individuals have been more inconsistent. Higher thyroid hormone levels have been correlated with poorer physical function (Ceresini et al., 2011; Simonsick et al., 2016) and frailty (Yeap et al., 2012) in older adults, supporting an association. In euthyroid individuals, a recent Korean study of over 36,000 euthyroid younger adults found that lean muscle mass, as measured by bio-impedance, was negatively associated with higher free triiodothyronine (FT3) but not with TSH or FT4. (Kwon et al., 2018). In contrast, higher T3 levels have been associated with better hand grip strength in two Chinese studies of middle aged and older adults. (Sheng et al., 2019; Gu et al., 2019).

Iatrogenic hyperthyroidism is the most common form of excess thyroid hormone. (Canaris et al., 2000; Mammen et al., 2015). In addition, older adults are more likely to be on thyroid hormone supplements such as levothyroxine (LT4). (Taylor et al., 2014). Furthermore, new appreciation for the physiologic changes in thyroid hormone regulation with aging and aging-related stressors has led to the recognition that many treated adults may have a relative excess of thyroid hormone even if the levels are within the reference range (Mammen, 2019). Therefore, understanding the relationship between thyroid hormone levels and body composition in older adults might support changes in the current goals of therapy when using thyroid hormone supplements in older adults to prevent the exacerbation and progression of sarcopenia.

This study tests the hypothesis that there are age and treatment related differences in the association between thyroid function test findings and lower limb lean muscle mass as measured by DEXA among community-resident older adults participating in the Baltimore Longitudinal Study of Aging (BLSA).

## Methods

The BLSA is an observational cohort study conducted by the Intramural Research Program of the National Institute on Aging (NIA) (Shock et al., 1984). Begun in 1958, the BLSA is the longest-running study of aging in the United States. DEXA scans and thyroid function tests are available from routine study visits since 2003. Serum for thyroid function tests is collected on the morning of the second day of a participant’s 3-day visit after an overnight fast. Those with acute illness are rescheduled and thyroid function tests were not analyzed for those requiring a home visit. Thyroid function tests (Thyroid Stimulating Hormone (TSH), FT4, Total T4, FT3, and Total T3) were performed for the BLSA in CLIA-certified laboratories. For these analyses, visits between January 2003 and the end of 2019 at which participants had TSH (reference range [0.5–4.7 mU/L in 2003–2004; 0.35–5.5 mU/L in 2005–2011; 0.4–4.0 mIU/L 2012 onwards) and FT4 (reference range [0.76–1.46 ng/dl]) within reference range were included. Laboratory values before 2011 were normalized to the current assay for comparative analyses (Mammen et al., 2017). Total body DEXA was performed using the Prodigy Scanner (General Electric) and analyzed with version 10.51.006 software. Both lower limbs were considered together for the purposes of this analysis. Sarcopenia was defined using the International Working Group on Sarcopenia DEXA cutoffs for Sarcopenia: Gait Speed < 1m/s along with appendicular Lean Mass (in kg)/Height (in m^2^) ≤ 7.23 for men, and ≤5.67 for women (Fielding et al., 2011). The current BLSA protocol was approved by the National Institutes of Health Intramural Institutional Review Board and all participants provide written informed consent.

### Statistical analysis

Pooled estimates from linear mixed-effects models to account for inter-participant variability were used on 5 imputed datasets to estimate the cross-sectional association between several thyroid function tests and DEXA measurements of lower-limb lean and fat mass measured in grams. Generalized linear mixed-effects models with logit links were used to estimate the association with each available thyroid function test. Estimates for all models were calculated for 1 mU/L difference in TSH, 1 pg/ml difference in FT3, 0.1 ng/dl difference in FT4, and 1 ug/dl difference in Total T4. All models were adjusted for concurrent LT4 use, age, race, sex, BMI, smoking history (past, current, former), history of alcohol intake (in the past 12 months), total cholesterol and systolic blood pressure. The models were also conducted stratified by age (≥65 vs. < 65 years old) or LT4 use. We used a False-Discovery Rate with an α of 0.05 to account for multiple comparisons (calculated separately for descriptive tables, linear mixed effect models, and generalized linear mixed effect models with logit link). Non-normality was detected for continuous variables using the Shapiro-Wilk test. Thus, Wilcoxon Rank-Sum tests for continuous variables and Chi-square or Fisher’s exact tests for categorical variables were used to compare baseline characteristics. Missing data patterns were assessed using Little’s test for Missing Completely at Random and were found to be significant (*p*-value <0.05). Therefore, multiple imputations with chained equations were conducted to account for missing covariate data (8.3%).

Analyses were conducted using the open-source R software version 4.2.0 (haven, dplyr, naniar, gtsummary, amelia, mitools, lme4, lmerTest, lattice; R Foundation for Statistical Computing). All linear mixed effect models were tested for linearity, homogeneity of variance, and normally distributed residuals assumptions.

## Results

One-thousand four hundred and forty-two participants made a total of 5306 eligible study visits (mean 3.68 visits per participant with 60% of participants having at least 3 visits). Participants had a median age of 68 years at their first qualifying visit (index visit), 50% were female, and 69% white (Table 1). LT4 use was present in 9.4%, and significantly more common among older adults compared to those younger than age 65 (12% v 6.4%, *p* < 0.001). Median gait speed was also found to be lower in older adults (1.1 vs. 1.3 m/s, *p*-value <0.001). In a fully adjusted linear mixed effect model with all participants, FT4 as negatively associated with lean mass (beta: −88.49; 95% Confidence Interval (CI): −122.78, - vs. 54.20; *p* < 0.001), but was not associated with fat mass, in the lower limbs (Table 2). The association between FT4 and lean mass was present only among older participants (beta: −66.79; 95% CI: −102.24, −31.33; *p* < 0.001) and was not observed in younger participants (beta: −60.60, 95% CI: −138.67, 17.47; p: 0.13), lean and fat mass were not associated with any of the thyroid function tests. Additionally, in fully adjusted generalized linear mixed effect models with logit link conducted on all participants, FT4 was associated with prevalent sarcopenia (OR: 1.11; 95% CI: 1.01, 1.22; *p*-value: 0.037). Trends in older adults indicate a similar propensity for sarcopenia with higher levels of FT4 (OR: 1.09; 95% CI: 1.01, 1.18; *p*-value: 0.039), though this was not the case for younger adults (Table 3). FT3 levels were positively associated with fat mass only among older adults (beta: 178.90; 95% CI: 17.55, 340.25; *p* < 0.001). These associations were no longer significant when analysis was limited to the 589 visits on LT4 (beta = −21.35; 95% CI: −105.64, 65.04; *p* = 0.62). TSH and Total T4 were not associated with body composition measures, and Total T3 failed to meet the models’ linear assumptions even post-imputation for missing values. Higher FT3:FT4 ratio was associated with higher lean muscle mass and was protective of sarcopenia risk in the full cohort, a finding consistent but no longer significant when restricted to older adults.

**TABLE 1:** Descriptive statistics of individuals at the index visit.

Characteristic	Full cohort N = 14,42^1^	Participants <65 years of age, N = 612^1^	Participants ≥65 years of age, N = 830^1^	*p*-value^2^
**Age (years)**	68.0 (58.0, 77.0)	57.0 (53.0, 61.0)	75.0 (70.0, 81.0)	<0.001
**Sex**				<0.001
Male	722/1,442 (50%)	354/612 (58%)	368/830 (44%)	
Female	720/1,442 (50%)	258/612 (42%)	462/830 (56%)	
**Race**				<0.001
White	998/1,439 (69%)	359/611 (59%)	639/828 (77%)	
Black	354/1,439 (25%)	206/611 (34%)	148/828 (18%)	
Other	73/1,439 (5.1%)	39/611 (6.4%)	34/828 (4.1%)	
Prefer Not to Say	7/1,439 (0.5%)	3/611 (0.5%)	4/828 (0.5%)	
Don’t Know	7/1,439 (0.5%)	4/611 (0.7%)	3/828 (0.4%)	
**BMI**	26.3 (23.8, 29.9)	27.1 (24.3, 31.0)	25.8 (23.6, 28.8)	<0.001
**Smoking History**				<0.001
Never	855/1,419 (60%)	397/602 (66%)	458/817 (56%)	
Quit >10 years ago	503/1,419 (35%)	169/602 (28%)	334/817 (41%)	
Quit ≤10 years ago	28/1,419 (2.0%)	16/602 (2.7%)	12/817 (1.5%)	
Current Smokers	33/1,419 (2.3%)	20/602 (3.3%)	13/817 (1.6%)	
**Alcohol (Past 12 mo)**				0.6
No	159/945 (17%)	71/434 (16%)	88/511 (17%)	
Yes	784/945 (83%)	363/434 (84%)	421/511 (82%)	
Prefer not to say	2/945 (0.2%)	0/434 (0%)	2/511 (0.4%)	
**Gait Speed (m/s)**	1.2 (1.1, 1.4)	1.3 (1.1, 1.4)	1.1 (1.0, 1.3)	<0.001
**Fat Mass, Both Legs (g)**	8,565.9 (6,334.9, 11,224.2)	9,359.0 (7,047.6, 12,204.6)	8,073.9 (5,933.3, 10,527.1)	<0.001
**Lean Mass, Both Legs (g)**	15,210.1 (12,558.0, 18,424.9)	15,418.6 (12,912.4, 19,220.3)	15,150.7 (12,328.2, 17,853.9)	<0.001
**TSH**	2.2 (1.5, 3.1)	2.1 (1.4, 2.8)	2.3 (1.6, 3.3)	<0.001
**Free T4**	1.0 (0.9, 1.2)	1.0 (0.9, 1.2)	1.0 (0.9, 1.1)	0.14
**Free T3**	2.9 (2.6, 3.2)	3.0 (2.7, 3.3)	2.8 (2.5, 3.1)	<0.001
**Total T4**	7.6 (6.7, 8.5)	7.6 (6.6, 8.7)	7.5 (6.7, 8.5)	0.3
**Total T3**	87.0 (78.0, 98.0)	89.5 (80.0, 102.0)	85.0 (74.5, 95.0)	0.032
**LT4 Use**				<0.001
No	1,306/1,442 (91%)	573/612 (94%)	733/830 (88%)	
Yes	136/1,442 (9.4%)	39/612 (6.4%)	97/830 (12%)	

**TABLE 2:** Association of Markers of Thyroid Function and Dual-Energy X-ray Absorptiometry measurements of the legs using Linear Mixed Effects Models*

	In visits of all participants (N = 5306 visits)			
Predictor	Estimates for Lean Mass [95% Confidence Interval]	*p*-value	Estimates for Fat Mass [95% Confidence Interval]	*p*-value
TSH^1^	52.72 [ −10.47, 115.91]	0.10	10.67 [ −48.77, 70.11]	0.81
Free T3^2^	41.06 [ −56.64, 138.76]	0.41	139.05 [ 31.60, 246.49]	0.01
Free T4^3^	−88.49 [ -122.78, -54.20]	<0.001	34.85 [1.51, 68.18]	0.04
Total T3	Models using Total T3 were not able to meet the linear models’ assumptions			
Total T4^4^	32.55 [ −1.41, 66.51]	0.06	48.77 [ 6.47, 91.11]	0.02
Free T3/Free T4 Ratio	163.10 [ 58.07, 268.13]	0.004	42.05 [-43.40, 127.50]	0.331
	Visits in which participants are aged ≥ 65 years old			
	Estimates for Lean Mass [95% Confidence Interval]	*p*-value	Estimates for Fat Mass [95% Confidence Interval]	*p*-value
TSH^1^	19.26 [ −43.29, 81.81]	0.54	−7.55 [ −62.80, 47.70]	0.60
Free T3^2^	96.25 [ −15.54, 208.03]	0.09	178.90 [ 17.55, 340.25]	<0.001
Free T4^3^	−66.79 [ -102.24, -31.33]	<0.001	49.47 [7.83, 91.11]	0.22
Total T4^4^	10.99 [ −27.99, 49.96]	0.58	47.16 [0.65, 93.66]	0.11
Free T3/Free T4 Ratio	161.98 [ 56.63, 270.33]	0.005	65.27 [-61.91, 192.46]	0.053
	Visits in which participants are aged < 65 years old			
	Estimates for Lean Mass [95% Confidence Interval]	*p*-value	Estimates for Fat Mass [95% Confidence Interval]	*p*-value
TSH^1^	169.76 [41.23, 298.28]	0.01	132.73 [-3.49, 268.95]	0.04
Free T3^2^	−66.20 [-516.67,384.29]	0.75	−25.99 [−376.32, 324.33]	0.96
Free T4^3^	−60.60 [-138.67, 17.47]	0.13	111.84 [28.89, 194.79]	0.02
Total T4^4^	84.25 [-6.88, 175.37]	0.07	112.78 [22.43, 203.14]	0.04
Free T3/Free T4 Ratio	6.29 [-240.04, 252.62]	0.96	−206.30 [−420.02, 7.42]	0.06

* All models were adjusted for Levothyroxine (LT4) use, age, race, sex, BMI, smoking (past, current, former) history, alcohol intake history (in the past 12 months), serum total cholesterol, and systolic blood pressure (all as reported in each visit).

^1^ For every 1 mU/L change.

^2^ For every 1 pg/ml change.

^3^ For every 0.1 ng/dl change.

^4^ For every 1 ug/dl change.

**TABLE 3:** Association of Markers of Thyroid Function and Dual-Energy X-ray Absorptiometry Defined Sarcopenia^+^ using Generalized Linear Mixed Effects Models with Logit Link*.

	In visits of all participants (N = 5306 visits)	
Predictor	Odds Ratio for Sarcopenia [95% Confidence Interval]	*p*-value
TSH^1^	0.97 [0.84, 1.13]	0.678
Free T3^2^	0.92 [0.71, 1.19]	0.508
Free T4^3^	1.11 [1.01, 1.22]	0.037
Total T4^4^	0.95 [0.87, 1.04]	0.226
Free T3/Free T4 Ratio	0.76 [0.62, 0.94]	0.010
	Visits in which participants are aged ≥ 65 years old	
	Odds Ratio for Sarcopenia [95% Confidence Interval]	*p*-value
TSH^1^	0.99 [0.86, 1.13]	0.829
Free T3^2^	0.91 [0.70, 1.20]	0.508
Free T4^3^	1.09 [1.01, 1.18]	0.039
Total T4^4^	0.96 [0.88, 1.06]	0.436
Free T3/Free T4 Ratio	0.78 [0.58, 1.06]	0.103
	Visits in which participants are aged < 65 years old	
	Odds Ratio for Sarcopenia [95% Confidence Interval]	*p*-value
TSH^1^	1.05 [0.74, 1.49]	0.776
Free T3^2^	0.83 [0.31, 2.22]	0.690
Free T4^3^	1.15 [0.86, 1.42]	0.287
Total T4^4^	0.82 [0.65, 1.04]	0.096
Free T3/Free T4 Ratio	0.68 [0.36, 1.27]	0.217

*All models were adjusted for Levothyroxine (LT4) use, age, race, sex, BMI, smoking (past, current, former) history, alcohol intake history (in the past 12 months), serum total cholesterol, and systolic blood pressure (all as reported in each visit).

^1^ For every 1 mU/L change.

^2^ For every 1 pg/ml change.

^3^ For every 0.1 ng/dl change.

^4^ For every 1 ug/dl change.

+Defined as gait speed < 1m/s and appendicular Lean Mass (in kg)/height (in m2) ≤ 7.23 for men, and ≤5.67 for women.

In addition to the adjustment of LT4 use, we also conducted analyses limited to visits in which there was no LT4 use (N = 4717). Similar observations as our main analyses were seen in the overall participant sample (Lean mass beta for Free T4: −101.47; 95% CI: −138.84, −64.11; *p* < 0.001) and older adults (Lean mass beta for Free T4: −89.59; 95% CI: −139.01, −40.16; *p* = 0.001) for DEXA mass measurements. Free T4 was also seen to be associated with sarcopenia in LT4 non-users visits in the overall sample (OR: 1.12; 95% CI: 1.01, 1.24; *p*-value: 0.044) and in older adults (OR: 1.11; 95% CI: 1.00, 1.22; *p*-value: 0.049). In analysis of visits with LT4 use (N = 589), TSH (beta: 106.91; 95% CI: 8.31, 205.51; *p*-value: 0.03) and Total T4 (beta: 94.80; 95% CI: 19.59, 170.02; *p*-value: 0.01) were associated with lean mass, and Total T4 was found to be associated with fat mass (beta: 103.43%, 95% CI: 3.38, 203.48; *p*-value: 0.04) for unadjusted *p*-values. These associations are not significant once we adjust our *p*-values for multiple comparisons.

## Discussion

Our study found that higher FT4 levels are negatively associated with lower leg lean mass and positively associated with the presence of sarcopenia in euthyroid older adults. Our results show an association between FT4 and lean mass in both overall and older adult participants (a 1 ng/dl higher FT4 corresponds to roughly 700–900 g lower lean mass), as well as FT4 and fat mass in older adult participants (a 1 ng/dl higher FT4 corresponds to roughly 1.8 kg higher fat mass). The inverse association with lean and fat mass is consistent with a shift in body composition away from more fit phenotypes in those with higher thyroid hormone levels. Though our results are limited to the lower limbs, we speculate that these associations may also hold true for the whole body, as suggested by the higher odds of sarcopenia with higher FT4 in the overall and older adult participant sample. This is highly consistent with our prior findings of poorer physical function in older adults with higher FT4 levels within the reference range. (Simonsick et al., 2016). LT4 use is a source of higher thyroid hormone level in those on therapy, but the association between FT4 and muscle mass was no longer significant when restricted to those on LT4, which may be a function of the lower sample size (and consequently decreased power). One possible explanation for this is that LT4 use changes the feedback relationship between TSH, FT4 and T3, which may alter the accuracy of FT4 as marker of functional thyroid hormone levels in those on treatment. Several studies at Oregon Health and Science University have examined the effects of LT4 treatment on body composition by DEXA. (Samuels et al., 2016; Samuels et al., 2017; Samuels et al., 2018). Interestingly, in all three studies changes in resting energy expenditure and fat mass could be attributed to variation in FT3 levels. Therefore, DEXA or bio-impedance-based monitoring may prove helpful as a secondary screening method for outcomes in populations at risk for a relative excess of thyroid hormone, including those taking supplemental with thyroid hormone.

Although our study accounts for within-participants repeated measures, we cannot establish causality. Longitudinal examination of the possible catabolic effects of thyroid hormone on muscle with a robust measure of muscle quality is important to establish whether excess or inappropriate thyroid hormone use can contribute to the development of sarcopenia. Moreover, physical activity often decreases in older adults and can negatively impact muscle mass. While our analyses were not adjusted for a metric of physical activity, all of the included visits were performed by participants living independently and capable of ambulation including performing the 6-m walk test. Hence, activity levels in older adults of this cohort do not include values in the disability range. An indirect causation pathway with thyroid hormone levels affecting energy and activity level and thus indirectly muscle mass is none-the-less plausible. In addition, the protective effect seen with higher FT3:FT4 ratios raises the possibility of reverse causality in the findings. During acute stress, the conversion of FT4 to FT3 is decreased, lowering the ratio, and leading to the hypothesis that during aging a similar lowering of the ratio might accompany the stress of declining health. Our cohort is not affected by euthyroid sick syndrome by protocol, but factors such as chronic inflammation that promote muscle loss could also cause changes in thyroid hormone metabolism. Prospective studies are needed to further elucidate these possibilities. Finally, while DEXA is superior to clinical measures of body composition (such as BMI, gross weight, or waist circumference) or bio-impedance as used in prior research, it is not as accurate as CT scans, which can provide more robust biomarkers of body composition (intermuscular vs. intramuscular fat, and subcutaneous fat) and muscle quality (derived from detailed cross-sectional areas of specific muscles). (Mohajer et al., 2022). Our results are nonetheless consistent with findings of other groups similarly investigating thyroid hormone levels and sarcopenia measures. With future studies, investigation of distinct patterns of thyroid hormone levels specially in older adults may help detect abnormal thyroid function affecting body mass from age-related physiological changes. (Mammen, 2019).

## Data Availability

Publicly available datasets were analyzed in this study. This data can be found here: https://www.blsa.nih.gov/.
